# Leukemia Cutis at the Diagnosis of Chronic Lymphocytic Leukemia: A Case Report

**DOI:** 10.7759/cureus.117281

**Published:** 2026-10-01

**Authors:** Imane Mouaine, Rida Fatima Ezzahra, Omar Halloumi, Houda Elkhayat, Salma Fares

**Affiliations:** 1 Hematology, Centre Hospitalier Universitaire Mohammed VI, Agadir, MAR; 2 Faculty of Medicine and Pharmacy of Agadir, Université Ibn Zohr, Agadir, MAR; 3 Clinical Hematology and Cellular Therapy, Centre Hospitalier Universitaire Mohammed VI, Agadir, MAR; 4 Hematology, Institut Jules Bordet, Hôpital Universitaire de Bruxelles (HUB), Bruxelles, BEL; 5 Hematology, Medical School of Agadir, Université Ibn Zohr, Agadir, MAR; 6 Hematology, Faculty of Medicine and Pharmacy of Agadir, Université Ibn Zohr, Agadir, MAR

**Keywords:** chronic lymphocytic leukemia (cll), cutaneous infiltration, cutaneous manifestations, leukemia cutis, skin biopsy

## Abstract

Chronic lymphocytic leukemia (CLL) is the most common leukemia in adults. Specific cutaneous infiltration by leukemic cells is rare and, in exceptional cases, can be the presenting manifestation of the disease. We report the case of a 73-year-old man referred for lymphocyte-predominant hyperleukocytosis associated with infiltrated erythematous plaques on the left leg. Clinical examination found no tumor syndrome. The blood count showed hyperleukocytosis (298,000/mm³), severe anemia (hemoglobin, 4.6 g/dL), and profound thrombocytopenia (5,000/mm³). Blood immunophenotyping revealed a B-lymphoid clone consistent with typical CLL. Skin biopsy showed an atypical dermal lymphoid infiltrate, and immunohistochemistry (CD5+, CD20+, CD23+) was consistent with specific cutaneous involvement by CLL. Immunochemotherapy with rituximab, cyclophosphamide, and dexamethasone was initiated, resulting in marked improvement of the skin lesions after four cycles of treatment. Specific cutaneous infiltration is a rare manifestation of CLL and can, exceptionally, be the presenting feature of the disease. This case highlights the value of performing a skin biopsy for any atypical skin lesion associated with lymphocytosis.

## Introduction

Chronic lymphocytic leukemia (CLL) is a malignant hemopathy characterized by the clonal accumulation of mature B lymphocytes [1]. Diagnosis is established by clonal B-cell lymphocytosis ≥5 × 10⁹/L persisting for at least three months, along with a characteristic immunophenotype. CLL follows a heterogeneous clinical course, ranging from indolent forms that require only monitoring to progressive forms that require treatment [2]. Cutaneous manifestations of CLL may be specific, reflecting direct infiltration of the skin by leukemic cells, or nonspecific, such as pruritus, pyoderma gangrenosum, cutaneous vasculitis, Sweet syndrome, or erythroderma. Leukemia cutis is a specific dermatological manifestation [3], though a rare one, of CLL, occurring in fewer than 5% of cases [4]. It results from infiltration of the superficial and deep layers of the dermis by leukemic cells [5]. We report a rare case of specific cutaneous infiltration occurring at the time of CLL diagnosis and discuss its diagnostic, prognostic, and therapeutic features. 

## Case presentation

A 73-year-old man with no significant medical history, including no recent infectious episodes or medication use, was referred to our clinical hematology department for evaluation of marked, lymphocyte-predominant hyperleukocytosis. One month before presentation, he had noticed the onset of skin lesions on the left leg, without associated symptoms, notably pruritus. On examination, the lesions consisted of confluent purpuric and ecchymotic areas surrounded by petechiae, measuring approximately 8 cm in greatest diameter, with ill-defined borders. Given the severe thrombocytopenia (5,000/mm³) and the presence of immune-mediated cytopenias, thrombocytopenic purpura was considered a major differential diagnosis. A skin biopsy was therefore performed on an infiltrated area distinct from the purpuric lesions, following platelet transfusion support, to confirm the specific nature of the cutaneous involvement (Figure 1).

**Figure 1:**
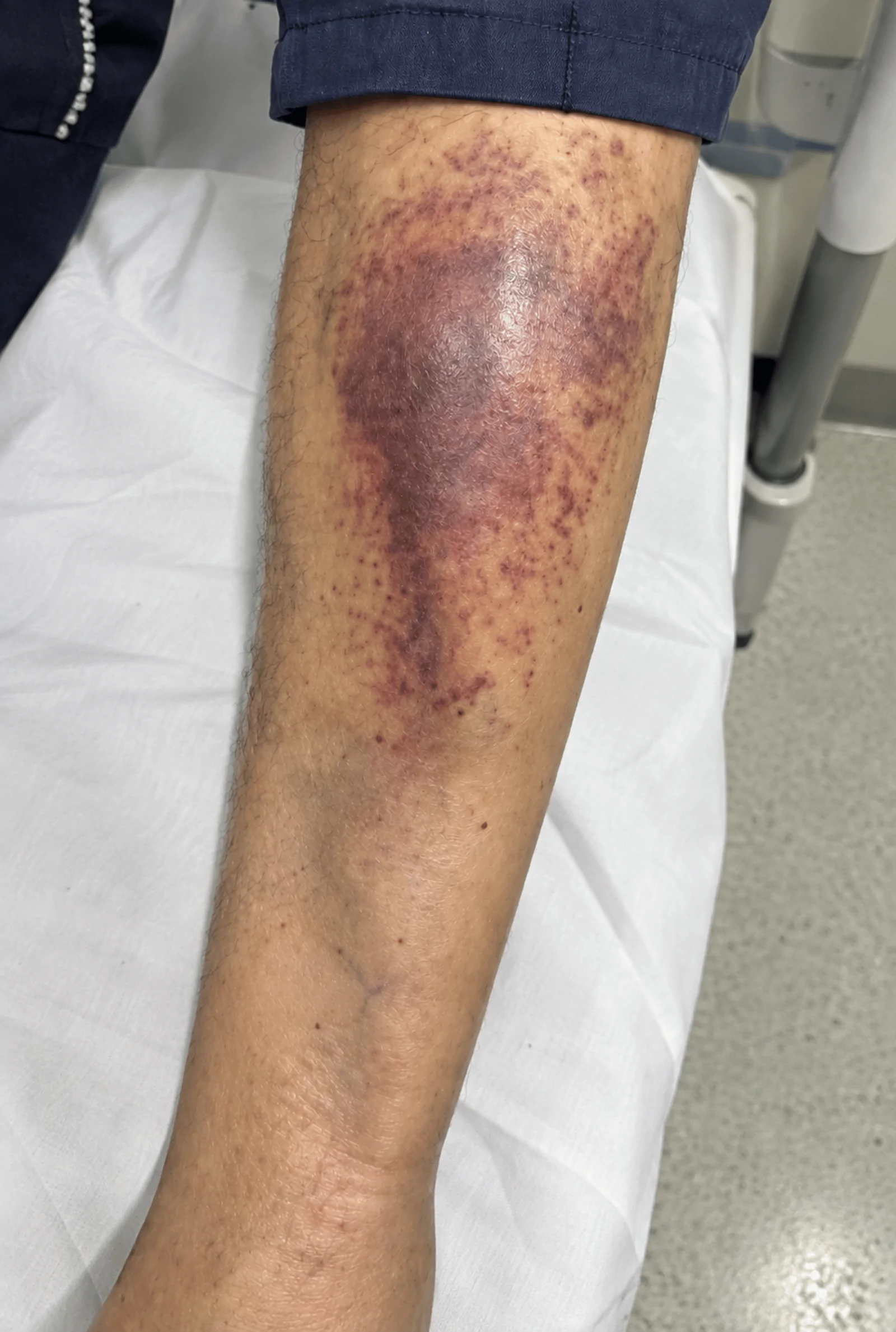
Infiltrated erythematous plaques on the left leg.

Clinical examination showed a WHO performance status of 3 [6]. There was no peripheral lymphadenopathy or hepatosplenomegaly. The overall clinical picture was one of general health deterioration.

The complete blood count revealed severe macrocytic, hypochromic anemia (hemoglobin, 4.6 g/dL; mean corpuscular volume (MCV), 111 fL; mean corpuscular hemoglobin concentration (MCHC), 26.7 g/dL), associated with profound thrombocytopenia (5,000/mm³) and marked hyperleukocytosis (298,000/mm³), predominantly due to lymphocytosis (251,700/mm³). The hemolysis workup was consistent with active autoimmune hemolysis, with a direct antiglobulin test (Coombs test) strongly positive at 37°C, specific for immunoglobulin G (IgG), with a positive irregular antibody screen. Haptoglobin was markedly decreased at 0.3 g/L, and lactate dehydrogenase (LDH) was elevated at 500 U/L. Total bilirubin was 30 mg/L, predominantly indirect (indirect fraction, 21 mg/L), and the reticulocyte count was elevated at 252,000/mm³, consistent with a regenerative hemolytic process. Vitamin B12 and folate levels were within normal limits, at 235 pg/mL and 15 ng/mL, respectively. Macrocytosis (MCV, 111 fL) was attributed to the associated reticulocytosis rather than to megaloblastic anemia (Table 1).

**Table 1:** Hematological parameters. The reference ranges provided in this table are based on standard clinical reference values. MCV, mean corpuscular volume; MCHC, mean corpuscular hemoglobin concentration; IgG, immunoglobulin G; LDH, lactate dehydrogenase

Parameter	Result	Reference range
Hemoglobin	4.6 g/dL	13.0-17.0 (men)/12.0-15.0 (women)
MCV	111 fL	80-100 fL
MCHC	26.7 g/dL	32-36 g/dL
Platelets	5,000/mm³	150,000-400,000/mm³
WBC	298,000/mm³	4,000-10,000/mm³
Lymphocytes	251,700/mm³	1,000-4,000/mm³
Reticulocyte count	252,000/mm³	25,000-100,000/mm³
LDH	500 U/L	140-280 U/L
Total bilirubin	30 µmol/L	5-17 µmol/L
Indirect (unconjugated) bilirubin	21 µmol/L	<13.7 µmol/L
Haptoglobin	0.3 g/L	0.3-2.0 g/L
Vitamin B12	235 pg/mL	200-900 pg/mL
Folate (vitamin B9)	15 ng/mL	3-17 ng/mL

Examination of the peripheral blood smear revealed a population of mature-appearing lymphoid cells, along with numerous Gumprecht shadows. Peripheral blood immunophenotyping identified a clonal B-cell population expressing CD19, CD20, CD5, CD23, and CD200, with lambda light-chain restriction, while FMC7 and CD79b were negative, yielding a Matutes score of 5.

The clinical picture was consistent with an Evans syndrome-like presentation, combining autoimmune hemolytic anemia and probable immune thrombocytopenia. In accordance with International Workshop on CLL (iwCLL) guidelines, autoimmune cytopenias should not be automatically equated with bone marrow failure for staging purposes, as doing so may lead to an overestimation of disease stage. Bone marrow examination was not performed, as the diagnosis of CLL had already been established with high confidence based on peripheral blood immunophenotyping (Matutes score of 5), and the clinical urgency posed by severe autoimmune cytopenias prioritized prompt treatment initiation. For the same reason, cross-sectional imaging (computed tomography (CT) scan or abdominal ultrasound) was not obtained.

The disease was accordingly classified as Binet stage C and Rai stage IV. Testing for TP53 abnormalities, IGHV mutational status, and 17p deletion by fluorescence in situ hybridization (FISH) was requested but could not be performed owing to limited resources and the unavailability of these assays at our institution.

Histological examination of the skin biopsy showed a regular epidermis with preserved architecture. The dermis was fibrous and hemorrhagic, containing a dense perivascular lymphoid infiltrate involving the superficial and deep dermis. This infiltrate was composed of small, atypical-appearing lymphocytes with irregular, often crushed, hyperchromatic nuclei, intermingled with more regular-appearing lymphocytes, findings consistent with cutaneous involvement by CLL. Immunohistochemistry confirmed tumor cell positivity for CD5, CD20, and CD23, with a Ki-67 proliferation index of approximately 20% (Figure 2).

**Figure 2:**
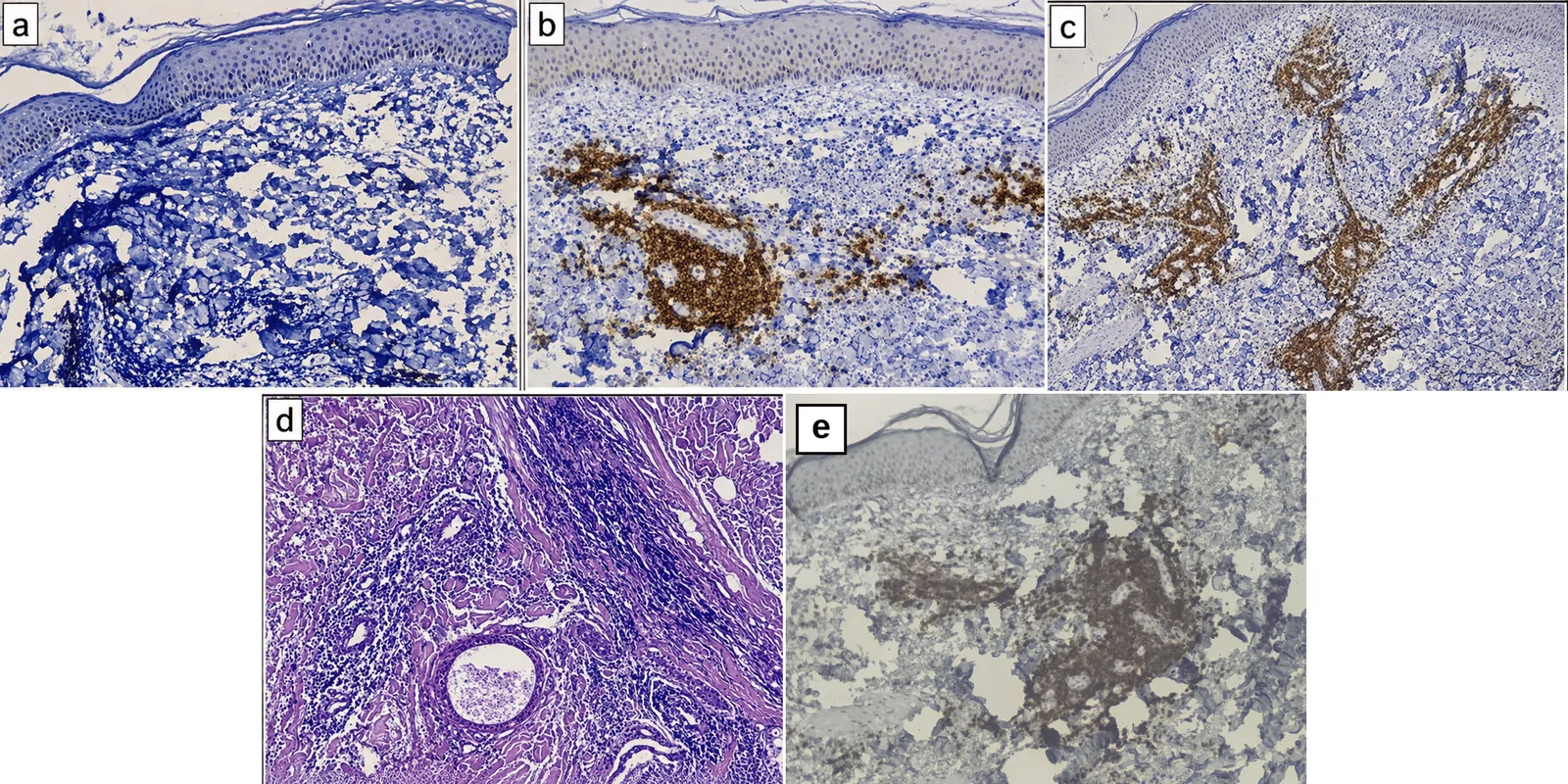
Histopathological examination of the skin biopsy shows (a) a Ki-67 index of approximately 20% (×200). Immunohistochemistry confirmed tumor cell positivity for (b) CD23 (×200), (c) CD20 (×200), and (e) CD5 (×100), with (d) a dense dermal lymphoid infiltrate on hematoxylin and eosin (H&E) staining (×200), consistent with cutaneous infiltration by chronic lymphocytic leukemia (CLL). H&E, hematoxylin and eosin; CD, cluster of differentiation; Ki-67, proliferation index

Treatment combining rituximab, cyclophosphamide, and dexamethasone was started at doses of 1,400 mg rituximab, 1,200 mg cyclophosphamide, and 20 mg dexamethasone per cycle. Because of the associated hemolytic anemia, the RCD regimen was chosen for a planned six cycles, with response assessment scheduled at the end of treatment.

Given the presence of severe autoimmune cytopenias requiring urgent disease control and the absence of access to TP53 mutation and fluorescence in situ hybridization (FISH) testing at our institution, immunochemotherapy combining rituximab, cyclophosphamide, and dexamethasone (RCD) was preferred over targeted agents such as Bruton tyrosine kinase (BTK) or B-cell lymphoma 2 (BCL2) inhibitors, which are increasingly used as first-line therapy in current CLL management. The RCD regimen was selected in particular for its demonstrated efficacy in controlling CLL-associated autoimmune cytopenias.

A corticosteroid prephase was administered prior to the first cycle. Treatment was then administered as follows: rituximab, 375 mg/m² intravenously on day 1 of cycle 1, increased to 500 mg/m² intravenously on day 1 from cycle 2 onward; cyclophosphamide, 250 mg/m²/day intravenously on days 1 to 3; and dexamethasone, 20 mg on days 1 to 3. Cycles were repeated every four weeks for a planned total of six cycles, with response assessment scheduled at the end of treatment.

Given the marked hyperleukocytosis (298,000/mm³), tumor lysis syndrome prophylaxis was instituted with hyperhydration and rasburicase. Hepatitis B virus (HBV) serology was performed prior to rituximab initiation and was negative. Prophylaxis against Pneumocystis jirovecii pneumonia was provided with trimethoprim-sulfamethoxazole, 800 mg three times weekly, and antiviral prophylaxis against herpes reactivation was provided with valaciclovir, 1 g daily. The patient also received red blood cell and platelet transfusion support in view of the severe anemia and thrombocytopenia.

After four cycles of chemotherapy, the outcome was favorable, with complete disappearance of the skin lesions and significant improvement in laboratory parameters: hemoglobin, 14 g/dL; white blood cell count, 8,900/mm³; neutrophils, 5,963/mm³; lymphocytes, 2,136/mm³; and platelets, 146,000/mm³ (Figure 3).

**Figure 3:**
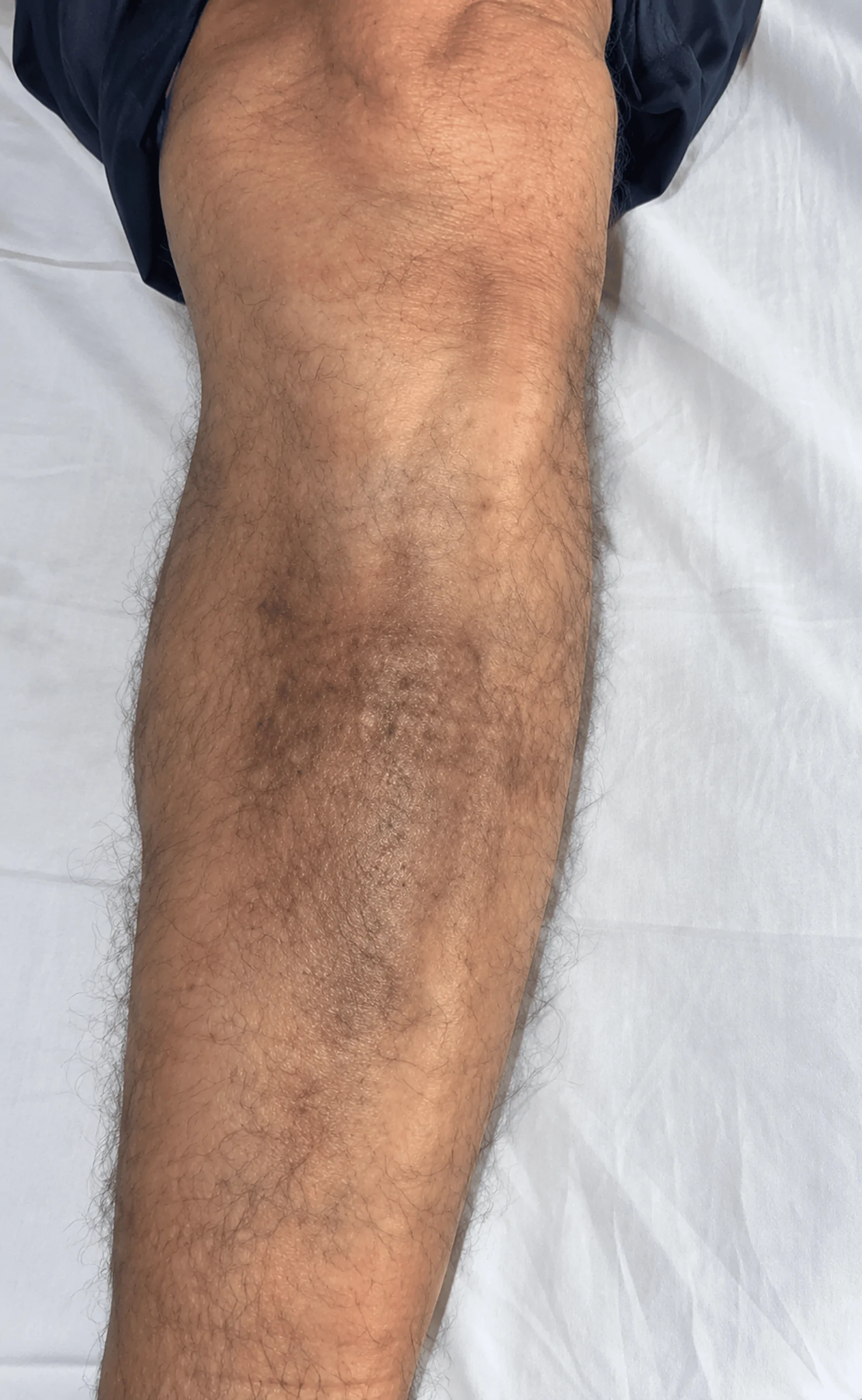
Disappearance of the skin lesions after four cycles of RCD. RCD, rituximab, cyclophosphamide, and dexamethasone

## Discussion

Cutaneous manifestations of CLL can be divided into two categories: specific lesions, resulting from direct infiltration of the skin by leukemic cells (leukemia cutis), and nonspecific lesions, secondary to treatment side effects, ineffective hematopoiesis, or paraneoplastic phenomena [7,8]. This distinction underscores the crucial role of histopathological examination in differentiating between the two entities.

Specific cutaneous infiltration may occur before, after, or concurrently with the diagnosis of CLL and can, exceptionally, represent its presenting feature, as observed in our case [7]. Such involvement remains a rare manifestation of CLL, and its occurrence as an inaugural presentation is exceptional [9].

In our patient, the biological workup revealed lymphocytosis associated with marked cytopenias. The Binet classification, which assesses disease prognosis based on hemoglobin and platelet levels together with the number of involved lymph node areas, does not take cutaneous involvement into account.

The pathogenic mechanism underlying leukemic skin infiltration remains poorly understood, although chemokine receptors and adhesion molecules, together with the genetic features of the leukemic clone, appear to play a significant role [10]. In particular, adhesion molecules such as integrins may contribute to the migration of leukemic cells toward the skin through selective skin-homing processes [10,11]. Cutaneous lymphocyte-associated antigen (CLA) and CC chemokine receptor 4 (CCR4), expressed on leukemic cells, can interact with E-selectin and/or TARC at the level of dermal postcapillary venules, thereby promoting the migration and anchoring of leukemic cells within the dermis. Some reports further suggest that leukemic cell infiltration occurs more frequently at sites with a prior history of skin infection or inflammation [12].

The independent prognostic value of specific cutaneous infiltration in CLL has not been established to date, owing to the rarity of reported cases and the heterogeneity of available data; this should not, however, be interpreted as evidence that cutaneous infiltration has no prognostic relevance. This uncertainty should not be conflated with the observation that skin involvement can occur across the full spectrum of CLL, including in patients with advanced-stage disease or aggressive biological features - as illustrated by our patient, who presented with Binet stage C disease and severe autoimmune cytopenias. In other words, leukemia cutis does not appear to independently worsen prognosis, but it may accompany, rather than cause, more advanced or biologically aggressive presentations of the disease. Cutaneous infiltrates are not currently incorporated into the Binet classification, which was developed and validated based on hemoglobin, platelet count, and lymph node involvement; their presence may nonetheless be taken into account, alongside other clinical and biological parameters, when the decision to initiate treatment is made [13].

It should nonetheless be noted that the vast majority of skin lesions observed in CLL are nonspecific [14]. Specific lesions resulting from leukemic cell infiltration remain uncommon among the cutaneous manifestations of CLL, occurring in less than 5% of patients [4], which is precisely why we chose to report this case.

The main clinical characteristics of cutaneous involvement reported in the literature, alongside those observed in our patient, are summarized in Table 2.

**Table 2:** Comparison of reported cutaneous manifestations of CLL with the current case. CLL, chronic lymphocytic leukemia; PS, performance status; WHO, World Health Organization

Feature	Lazarian et al. (2021) [15]	Aldapt et al. (2021) [16]	This case (2026)
Distribution pattern	Diffuse (*n* = 16), localized (*n* = 13), or regional (*n* = 9), among 38/46 cases with complete morphological description	Not specified as diffuse/localized/regional	Localized
Most affected site	Head and neck - most common site (22/38 cases), with earlobe involvement in 26% (10/38)	Head and neck, 19/56 (33.9%)	Left leg
Other sites	Arms and legs (17), trunk (13), other extremities (14)	Trunk and extremities, 15/56 (26.8%); isolated extremities, 11/56 (19.6%)	None
Lesion type/appearance	Papular and/or nodular (*n* = 24), patches (*n* = 9)	Papulonodular, 28/56 (50%); erythematous patches, 15/56 (26.8%)	Infiltrated erythematous plaques
Uncommon lesion types	Plaques, vesicles, mosquito-bite-like, rhinophyma-like, ulcerations, necrotic lesions	Ulceration/erosion uncommon, 3/56 (5.4%)	None
Associated symptoms	Not specified	Not specified	None (notably no pruritus)
Notable association	Infiltrates at sites of prior scars (herpes, mosquito bite, plant wound) in 4 patients	Skin involvement at sites of prior herpetic lesions, 5/56	None
Median time from skin lesion onset to CLL diagnosis/relapse	5.4 years (range 1-18 years)	5.5 years (range 1-21 years)	-
CLL stage at skin involvement	Binet A: 55%/B: 29%/C: 14% (*n* = 46)	Early stage: 17/56 (30.4%); advanced stage: 7/56 (12.5%); missing: 32/56 (57.1%)	Binet C
Performance status	Not specified	Not specified	WHO PS 3
Lymphadenopathy/organomegaly	Not specified	Not specified	None
Other notable finding	Two patients had Richter transformation; complete skin response in 74% (23/31) after systemic treatment	77.8% responded to treatment (25 CR, 10 PR)	General health deterioration

Lazarian et al. [15] reported that, among the 38 of 46 patients with a complete morphological description, skin lesions were diffuse (*n* = 16), localized (*n* = 13), or regional (*n* = 9). The head and neck region was the most frequently affected site (22/38 cases), with earlobe involvement in 26% of cases (10/38). Lesions were also reported on the arms and legs (*n* = 17), trunk (*n* = 13), and other extremities (*n* = 14).

Regarding lesion appearance, the most common cutaneous manifestations were papular and/or nodular (*n* = 24) and patches (*n* = 9). Plaques, vesicles, mosquito-bite-like lesions, rhinophyma-type lesions, ulcerations, and necrotic lesions were uncommon.

The median interval between the onset of skin manifestations and the diagnosis of CLL was 5.4 years (range, 1-18 years). Most patients were at an early stage of CLL when the skin lesions appeared (Binet stage A, 55%; stage B, 29%; stage C, 14%; *n* = 46), although two patients had undergone Richter transformation.

According to Aldapt et al. [16], among 56 reported cases, the head and neck region was the most commonly affected site (19/56, 33.9%), followed by the trunk and extremities (15/56, 26.8%) and isolated extremities (11/56, 19.6%). Papulonodular lesions were the most frequent clinical presentation (28/56, 50%), followed by erythematous patches (15/56, 26.8%); ulceration was uncommon (3/56, 5.4%). Cutaneous involvement at sites of prior herpetic lesions was reported in 5 cases. The median time to relapse with skin involvement was 5.5 years (range, 1-21 years). Among the 24 patients with available staging data, 17 were diagnosed at an early stage and 7 at an advanced stage, while staging data were missing for the remaining 32 patients.

The differential diagnosis of CLL skin lesions can be challenging, as their clinical presentation is highly variable and may mimic a number of other conditions depending on the appearance, number, and location of the lesions.

In the presence of isolated papules or nodules, cutaneous metastasis from a visceral tumor should be considered, as such metastases typically present as firmer or even very hard lesions. Purpuric or hemorrhagic nodules or plaques, particularly on the lower limbs, may raise the possibility of vasculitis or Kaposi sarcoma. Similarly, infiltrated or nodular leg lesions can sometimes resemble erythema nodosum. Depending on their morphology and degree of infiltration, erythematous or brownish-red plaques may also be mistaken for erythema multiforme, panniculitis, or mycosis fungoides [7].

Modern management of CLL has been transformed by the advent of targeted agents, notably BTK inhibitors (ibrutinib, acalabrutinib, zanubrutinib) and the BCL2 inhibitor venetoclax, which have become central to first-line therapy, particularly in patients with high-risk genetic features such as TP53 alteration, and whose use is largely guided by TP53 mutation and FISH status [17,18]. Rituximab is generally not combined with BTK inhibitors, as randomized trials have shown no additional benefit in progression-free or overall survival when rituximab is added to ibrutinib [19,20].

The presence of cutaneous involvement does not, in itself, dictate a specific systemic treatment strategy: management of CLL presenting with leukemia cutis follows the same general principles as CLL management as a whole, with treatment initiation guided primarily by systemic disease burden, cytopenias, and disease-related symptoms, while the skin lesions themselves typically respond to systemic therapy without requiring a distinct therapeutic approach [15].

In our patient, a BTK inhibitor- or venetoclax-based regimen was not used, mainly because TP53 mutation and FISH testing were unavailable at our institution, which precluded formal risk stratification, and because the severity of the associated autoimmune cytopenias called for a regimen with a well-established track record in this specific setting. We therefore favored immunochemotherapy combining rituximab, cyclophosphamide, and dexamethasone, given its demonstrated efficacy in controlling CLL-associated autoimmune cytopenias [21]. This choice, which reflected local resource constraints rather than an optimal risk-adapted strategy, nonetheless led to a favorable clinical and biological outcome, including complete resolution of the skin lesions. It is worth noting, however, that targeted agents, particularly BTK inhibitors, have also been reported to control CLL-associated autoimmune cytopenias effectively and could represent a valid alternative where such testing and agents are available [22].

For patients with isolated or localized cutaneous involvement not otherwise requiring systemic treatment, local approaches such as radiotherapy may also be considered, with complete responses reported in the majority of cases in published series [15].

## Conclusions

Cutaneous manifestations of CLL remain rare and can pose a genuine diagnostic challenge, sometimes presenting as infiltrated plaques that mimic other dermatological conditions and complicate the differential diagnosis. Establishing the diagnosis therefore relies on a multidisciplinary approach combining clinical, histological, and immunohistochemical findings.

This case underscores the value of careful dermatological assessment in patients with clonal B-cell lymphocytosis and suggests that skin biopsy should be strongly considered whenever cutaneous lesions appear clinically suspicious for specific leukemic infiltration.

## References

[1] Raufi A, Alsharedi M, Khelfa Y, Griswold DC, Lebowicz Y (2016). Leukemia cutis in a patient with chronic lymphocytic leukemia presenting as bilateral helical nodules. SAGE Open Med Case Rep.

[2] Rai KR, Jain P (2016). Chronic lymphocytic leukemia (CLL)-then and now. Am J Hematol.

[3] Pedreira-García WM, Nieves D, Montesinos-Cartagena M, Cortés CA, Cáceres-Perkins W, Rabelo-Cartagena J (2022). Leukemia cutis as the initial manifestation of chronic lymphocytic leukemia progression. Cureus.

[4] Chang YW, Lee CH, Tseng HC (2021). Leukemia cutis in a medical center in southern Taiwan: a retrospective study of 42 patients. J Formos Med Assoc.

[5] Osmola M, Gierej B, Waszczuk-Gajda A (2019). A rare manifestation of chronic lymphocytic leukaemia - leukaemia cutis treated with ibrutinib. Medycyna Paliatywna.

[6] Oken MM, Creech RH, Tormey DC, Horton J, Davis TE, McFadden ET, Carbone PP (1982). Toxicity and response criteria of the Eastern Cooperative Oncology Group. Am J Clin Oncol.

[7] Wagner G, Fenchel K, Back W, Schulz A, Sachse MM (2012). Leukemia cutis - epidemiology, clinical presentation, and differential diagnoses. J Dtsch Dermatol Ges.

[8] Szukalski Ł, Sysakiewicz-Buda M, Mêciñska-Jundziłł K, Białecka A, Czyż J, Czajkowski R (2019). Cutaneous manifestation of chronic lymphocytic leukemia: a case report and a literature review. Postepy Dermatol Alergol.

[9] Hallek M, Al-Sawaf O (2021). Chronic lymphocytic leukemia: 2022 update on diagnostic and therapeutic procedures. Am J Hematol.

[10] Cho-Vega JH, Medeiros LJ, Prieto VG, Vega F (2008). Leukemia cutis. Am J Clin Pathol.

[11] Bakst R, Powers A, Yahalom J (2020). Diagnostic and therapeutic considerations for extramedullary leukemia. Curr Oncol Rep.

[12] Haidari W, Strowd LC (2019). Clinical characterization of leukemia cutis presentation. Cutis.

[13] Vonarx M, Carpentier O, Cazin B, Bouchindhomme B, Delaporte E (2012). Chronic lymphocytic leukaemia presenting with specific cutaneous infiltrates in the absence of lymphocytosis: two cases. Ann Dermatol Venereol.

[14] Zhao X, Li T, Wang P, Bai X, Cheng J (2022). Diagnosis and prognostic analysis of skin infiltration in chronic lymphocytic leukemia: a case report and literature review. Transl Cancer Res.

[15] Lazarian G, Munger M, Quinquenel A (2021). Clinical and biological characteristics of leukemia cutis in chronic lymphocytic leukemia: a study of the French innovative leukemia organization (FILO). Am J Hematol.

[16] Aldapt MB, Yassin M (2021). Leukemia cutis as an early presentation or relapsing manifestation of chronic lymphocytic leukemia (CLL). Acta Biomed.

[17] Hampel PJ, Parikh SA (2022). Chronic lymphocytic leukemia treatment algorithm 2022. Blood Cancer J.

[18] Robak T, Witkowska M, Smolewski P (2022). The role of Bruton's kinase inhibitors in chronic lymphocytic leukemia: current status and future directions. Cancers (Basel).

[19] Ahn IE, Tian X, Wiestner A (2020). Ibrutinib for chronic lymphocytic leukemia with TP53 alterations. N Engl J Med.

[20] Sharman JP, Egyed M, Jurczak W (2022). Efficacy and safety in a 4-year follow-up of the ELEVATE-TN study comparing acalabrutinib with or without obinutuzumab versus obinutuzumab plus chlorambucil in treatment-naïve chronic lymphocytic leukemia. Leukemia.

[21] Rossignol J, Michallet AS, Oberic L (2011). Rituximab-cyclophosphamide-dexamethasone combination in the management of autoimmune cytopenias associated with chronic lymphocytic leukemia. Leukemia.

[22] Noto A, Cassin R, Mattiello V, Reda G (2021). The role of novel agents in treating CLL-associated autoimmune hemolytic anemia. J Clin Med.

